# Self-reported physical and emotional health among left behind children in Lithuania. A pilot study

**DOI:** 10.1093/eurpub/ckac131.433

**Published:** 2022-10-25

**Authors:** J Racaite, K Antia, V Winkler, E Dambrauskaite, I Tracevskyte, S Lesinskiene, G Surkiene

**Affiliations:** 1 Department of Public Health, Faculty of Medicine, Vilnius University, Vilnius, Lithuania; 2 Heidelberg Institute of Global Health, Heidelberg University Hospital, Heidelberg, Germany; 3 Institute of Psychology, Faculty of Philosophy, Vilnius University, Vilnius, Lithuania; 4 Clinic of Psychiatry, Faculty of Medicine, Vilnius University, Vilnius, Lithuania

## Abstract

**Background:**

Left behind children (LBC) are children living with caregivers, while their parents work abroad. In recent decades Lithuania had lost ¼ of its population due to emigration, but the prevalence of LBC and their health needs are not sufficiently studied. The aim of study was to evaluate the association between self-reported health, emotional and behavioural difficulties between LBC and non-LBC.

**Methods:**

The cross-sectional study was approved by the Biomedical Research Ethics Committee, No. 2021/11-1378-861. In March 2022, this pilot study collected data from adolescents aged 12-17 years at three randomly selected schools from the migration affected regions. Parents and children provided informed consent to participate in pilot study. Self-reported measures collected from the participants: Strengths, and Difficulties Questionnaire (Goodman, 2005) and a questionnaire on demographics and on the health situation. Chi^2^ tests and logistic regression were calculated by using Stata (version 15.1).

**Results:**

The sample consisted of 127 children (mean age 15.2, SD 1.27; 54 boys, 72 girls) including 39 LBC. Mostly fathers left to work abroad (n = 36), but majority of children (n = 36) regularly communicated remotely with their migrated parents. Binary logistic regression results show that LBC children tend to evaluate their health as ‘poor, ‘bad', or ‘very bad’ (OR 2,33; 95CI [1,02-5,33]). There were no differences for emotional and behavioural problems. Preliminary results from multiple regression model showed that children with self-reported emotional/behavioural difficulties (OR 3,06; 95CI [1,12-7,84]), females (OR 4,99;95CI [1,55-16,13]) and LBC (OR 2,44; 95CI 0,95-6,25]) more likely evaluate their health as ‘poor', ‘bad’ or ‘very bad’ (likelihood-ratio test=23,72; model p < 0.001).

**Conclusions:**

This pilot study found negative association between parental migration and children’s self-reported health. However, a more comprehensive study with a larger sample size is needed.

**Key messages:**

• Pilot study suggests negative association between parents migration and children’s self-reported health.

• More comprehensive study in bigger sample for the emotional/behavioural/communication difficulties between left behind children needs to be done.

